# Supplementary material for: Estimated burden, and associated factors of Urinary Incontinence among Sub-Saharan African women aged 15–100 years: A systematic review and meta-analysis
Source: PLOS Glob Public Health. 2022 Jun 2;2(6):e0000562. doi: 10.1371/journal.pgph.0000562 (PMC10021416; doi:10.1371/journal.pgph.0000562)
Supplement: S1 Table — (DOCX) [file pgph.0000562.s002.docx]

S1 Table: Search strategies for the individual database

PubMed N=1392

| Search | Search term |
| --- | --- |
|  | Prevalence OR burden OR incidence |
|  | Incontinence OR Urinary incontinence OR bladder control OR non-fistula incontinence OR fecal incontinence OR bowel control OR pelvic organ prolapse OR utero-vaginal prolapse. |
|  | \| Angola OR Benin OR Botswana OR Burkina Faso OR Burundi OR Cameroon OR Cape Verde OR Central African Republic OR Chad OR Comoros OR Congo OR Cote d'Ivoire OR Djibouti OR Equatorial Guinea OR Ethiopia OR Gabon OR The Gambia OR Ghana OR Guinea OR Guinea-Bissau OR Kenya OR Lesotho OR Liberia OR Madagascar OR Malawi OR Mali OR Mauritania OR Mauritius OR Mozambique OR Namibia OR Niger OR Nigeria OR Rwanda OR Sao Tome and Principe OR Senegal OR Seychelles OR Sierra Leone OR Somalia OR South Africa OR Sudan OR Swaziland OR Tanzania OR Togo OR Uganda OR Zaire OR Zambia OR Zimbabwe \| \| --- \| |
|  | Free Full text, observational study, from 2000/1/1 to 2021/9/30, Humans, English, Female |
|  | 1 AND 2 AND 3 AND 4 |

Google scholar N=417

| Search | Search term |
| --- | --- |
|  | Prevalence OR burden OR incidence |
|  | Incontinence OR Urinary incontinence OR bladder control OR non-fistula incontinence OR fecal incontinence OR bowel control OR pelvic organ prolapse OR utero-vaginal prolapse. |
|  | ‘’Sub-saharan Africa’’ |
|  | 2000 to 2021, sort by relevance, any type |
|  | 1 AND 2 AND 3 AND 4 |

AJOL N=120

| Search | Search term |
| --- | --- |
|  | Prevalence OR burden OR incidence |
|  | Incontinence OR Urinary incontinence OR bladder control OR non-fistula incontinence OR fecal incontinence OR bowel control OR pelvic organ prolapse OR utero-vaginal prolapse. |
|  | ‘’Sub-saharan Africa’’ |
|  | 2000 to 2021, sort by relevance, any type |
|  | 1 AND 2 AND 3 AND 4 |
