# Supplementary material for: Estimated burden, and associated factors of Urinary Incontinence among Sub-Saharan African women aged 15–100 years: A systematic review and meta-analysis
Source: PLOS Glob Public Health. 2022 Jun 2;2(6):e0000562. doi: 10.1371/journal.pgph.0000562 (PMC10021416; doi:10.1371/journal.pgph.0000562)
Supplement: S2 Table — (DOCX) [file pgph.0000562.s003.docx]

**S2 Table:** Risk of bias/quality assessment

| Study ID | Selection | Comparability | Outcome |  |
| --- | --- | --- | --- | --- |
| Berhe et al.(13) | **** | ** | *** | Low |
| Beketie et al.(14) | ***** | ** | *** | Low |
| Ofori et al.(15) | **** | ** | *** | Low |
| Adanu et al.(42) | **** | ** | *** | Low |
| Balde et al.(44) | **** | * | *** | Low |
| Bekele et al.(45) | **** | ** | *** | Low |
| Bowling et al.(25) | *** | * | ** | Moderate |
| Ojengbede et al.(34) | **** | ** | ** | Low |
| Usifoh et al.(37) | **** | ** | ** | Low |
| Rabiu et al.(29) | ******* | ****** | ******* | Moderate |
| Okunola et al.(35) | ******** | ****** | ******* | Low |
| Abiola et al.(40) | *** | * | *** | Low |
| Akinlusi et al.(16) | *** | ** | *** | Low |
| Adaji et al.(41) | *** | * | ** | Moderate |
| Yağmur et al.(39) | *** | * | ** | Moderate |
| Badejoko et al.(43) | **** | ** | *** | Low |
| Bello et al.(36) | **** | * | *** | Low |
| Njoku et al.(32) | **** | ** | *** | Low |
| Irshad et al.(28) | *** | * | ** | Moderate |
| Obioha et al.(33) | *** | ** | ** | Moderate |
| Gashugi et al.(26) | *** | * | *** | Low |
| Patel et al.(27) | *** | * | ** | Moderate |
| Skaal et al(37) | **** | ** | *** | Low |
| Madombwe et al.(30) | **** | * | *** | Low |
| Masenga et al.(31) | **** | ** | *** | Low |
