# Supplementary material for: Estimated burden, and associated factors of Urinary Incontinence among Sub-Saharan African women aged 15–100 years: A systematic review and meta-analysis
Source: PLOS Glob Public Health. 2022 Jun 2;2(6):e0000562. doi: 10.1371/journal.pgph.0000562 (PMC10021416; doi:10.1371/journal.pgph.0000562)
Supplement: S3 Table — (DOCX) [file pgph.0000562.s004.docx]

**S3 Table:** Meta-regression of burden of UI in SSA women by sample size and year of publication

| Variable | Meta-regression | |
| --- | --- | --- |
|  | Coefficient | p-value |
| Sample size | -0.0001 | 0.048 |
| Year of publication | -0.0033 | 0.533 |
