# Supplementary material for: Estimated burden, and associated factors of Urinary Incontinence among Sub-Saharan African women aged 15–100 years: A systematic review and meta-analysis
Source: PLOS Glob Public Health. 2022 Jun 2;2(6):e0000562. doi: 10.1371/journal.pgph.0000562 (PMC10021416; doi:10.1371/journal.pgph.0000562)
Supplement: Attachment — Submitted filename: response to reviewers.docx [file pgph.0000562.s005.docx]

**Responses to Reviewers Comments**

I humbly extend my warmest greetings to you and your reputable journal for your swift responses and feedback. I write on behalf of my colleague authors to submit our responses to the invaluable comments provided by your astute experienced reviewer. The format employed follows a point-to-point mode of reply to all comments. Please find below our responses:

**Reviewer 1**

**Comment:** Page 5: “Hospital and community based-studies had been conducted for UI in SSA countries.” - Please provide a reference for this statement.

**Response:** The authors would like to thank the reviewer for this observation. The statement is now referenced as recommended.

**comment:** Inclusion criteria: Some sentences are incomplete. For example: “Original observational studies published in English. An adult SSA woman [≥18 years].” - these two sentences are incomplete and grammatically incorrect.

**Response:** Thanks for the comment. The incomplete sentences have now been corrected and modified. The sentence now reads ‘’ Original observational studies published in English and adult SSA woman aged ≥18 years were included’’

**Comment:** Exclusion criteria: It started with an incomplete statement. For example: “Studies reporting animal studies, reviews, commentaries, letter to editors.”

**Response:** Thanks for the comment. The incomplete sentences have now been corrected and modified. The sentence now reads ‘’Studies reporting animal studies, reviews, commentaries, letter to editors were excluded’’.

**Comment:** Search: “The search was limited to January, 2000- September, 2021.” It means the search timeline was between January 2020 to September 2021 which is not correct, I guess. Did the author consider articles published during this time? Or, is this the timeframe of searching the databases. If the authors considered the timeline from inception to September 2021, please mention that clearly.

**Response:** Thanks for the comment and the observation. The authors considered articles published within this time frame. For clarity, we have revised the sentence. It now reads ‘’ The articles that were considered were published between 2000/1/1 and 2021/9/30’’

**Comment:** Database: Google scholar is a search engine, not a database. Africa Journal Online is a local repository. Authors should search SCOPUS, Web of Science, EMBASE (at least two of these three) in addition to Medline.

**Response:** The authors would like to thank the reviewer for the suggestion and recommendation. However, the authors do not free access to the above-named databases. As a results, the authors have taken cognizance of limitation in our study. Which reads ‘’ Finally, the authors identified relevant studies using a small database’’. Nonetheless, this is the first and largest systematic review and meta-analysis on the burden of UI among SSA women. Our results are more reliable evident by no obvious publication bias

**Comment:** A comprehensive search strategy for the Medline search should be provided. The search strategy for other databases should be provided as a supplementary file. As per the PRISMA 2020 guidelines, a search strategy for all the databases should be provided.

Response: Thanks for the comment. The authors have now provided the comprehensive search strategies for all databases in supplementary file

**Comment:** The current search strategy provided by the authors is revealing 618,343 articles (searched on 25 January 2022) from Medline/Pubmed. I don’t know how the authors have got only 720 articles from Medline/Pubmed. Can you please explain?

**Response:** The authors would like to thank the reviewer for this observation. First of all, it was oversight on the part of the authors. The current revised search strategy from 2000/1/1 to 2021/9/30 revealed 1392 articles in PubMed. However, no new eligible article was found to be included. Hence, the final eligible articles still remain 25.

**Comment:** Selection process: “To ensure a rigorous review strategy, any duplicate articles were reviewed individually by two independent co-authors [MA, and KA,] in a double blinded process, and then rejected before selecting a unique collection of papers for this study” - What do you mean by “any duplicate article”? Two independent authors should screen all the articles.

Response: Thanks for the comment. The sentence has been revised and modified for clarity. It reads ‘’ To ensure a rigorous review strategy, articles were reviewed individually by two independent co-authors [MA, and KA,] ‘’

**Comment:** The screening of articles is done in two phases. At first the title abstract screening, and then the full-text screening. Both the stages should be performed by two independent review authors independently. It should be mentioned properly.

**Response:** Thanks for the comment. The sentence modified for clarity. Which reads ‘’ The data screening was done in two stages; the title abstract screening, followed by the full-text screening. Both steps were completed independently by two review authors.’’

**Comment:** Results: Study selection: Authors have removed 550 articles even before the screening! This is strange. They should mention the specific reason for the pre-screening exclusion. The authors should mention the causes of exclusion of 175 articles that underwent full-text screening.

**Response:** Thanks for the comment. Figure 1 has been modified based on the revised search strategy. Also, the cause of exclusion has been explained in Figure 1.

**Comment:** Characteristics of the included studies and quality assessment: “Nigeria had the highest number of eligible studies [n=14], followed by Ethiopia [n=3], followed by Ghana and South Africa with two studies each.” - This calculation is showing 21 articles. Initially, the authors mentioned that they have included 25 articles.

**Response:** Thanks for the comment. This is so because, the other countries such as Guinea, Sierra Leone, Tanzania, and Liberia with one study each were not mentioned. However, those studies can be found in Table 1.

**Comment:** Please add a paragraph stating the research implication. What are the recommendations for conducting further research based on the findings of this systematic review? What are the policy implications of the findings?

**Response**: Thanks for the comment. The authors have incorporated the above suggestion in the work. It reads ‘’ Our research showed that, at least 21% of women in SSA have some form of urinary incontinence. This implies that there is the need to increase public health education and create awareness among people in order to promote health seeking behaviors among women. Healthy practices can be encouraged among women in order to reduce the disease burden in women. Such practices may include; maintaining a healthy body weight through exercise and diet to reduce the incidence of obesity and its complications. Avoiding constipation by taking in a high fiber diet, adequate intake of water, reducing immobility and regular emptying of bowel. Women should be taught how to strengthen their pelvic floor muscles especially during and after pregnancy by performing kegel’s exercises. Family planning methods should be made accessible to all irrespective of their social and economic status so women can effectively exercise their rights to choose the number of children they would want to have while taking into consideration their health. Finally, there are few studies examining the burden of UI, particularly in SSA's eastern and southern regions. It is therefore critical that more researches be undertaken in these sub-regions in order to obtain a complete, accurate, and consistent picture of this treatable condition’’.

**Comment:** The authors included articles published in English only? Isn’t it a limitation?

Response: Thanks for the comment. We have taken cognizance of this limitation and accordingly added. It reads ‘’ Furthermore, only articles published in English were considered’’.

**Comment:** Overall, there should be thorough language editing.

Response: Thans for the comment. The entire manuscript has been thoroughly read once again by all authors and a third independent editor to correct all grammatical errors which addresses the grammatical concerns raised by the reviewer

Reviewer 2

Introduction:
**Comment:** The introduction needs reorganization. The first paragraph may mislead the readers regarding the target topic. I would suggest focusing on UI in the opening paragraph, keeping only one or two sentences regarding pelvic floor muscles and pelvic floor disorders

**Response:** Thanks for the comment. First paragraph modified accordingly as recommended by the reviewer.
**Comment:** It would be great if the authors could provide reference of some low-and-middle income countries also, which are comparable to the SSA countries

Response: Thanks for the comment. Authors have now provided reference from LMICs. it reads’’ A comprehensive review and meta-analysis of 54 studies comprising 138722 women aged 10 to 90 years in Low- and Middle-Income Countries estimated the burden of UI to be 26%. In addition, the UI ranged from 2.8 % in Nigeria to 57.7% in the Islamic Republic of Iran(11)
**Comment:** The 2nd paragraph needs reorganization and resequencing of the sentences. Here, the authors provided definition and subtypes, followed by consequences. Then, all on a sudden, they provided US data, which seem irrelevant in the context

**Response:** Thanks for the comment. The last sentence of the 2^nd^ paragraph is removed.
**Comment**: The notion regarding limitation of underestimation of the studies conducted in SSA has not been references

**Response:** Thanks for the comment and observation. The sentence is now referenced
**comment:** The rationale of doing a systematic review should be at the last paragraph

**Response:** Thanks for the comment. The rationale of doing a systematic review is now moved to the last paragraph.

Research question
**Comment:** Should be described in narrative instead of bullets

**Response:** Thanks for the comment. The research questions are now described in narrative form

Methods
**comment:** It is great that the review is PROSPERO registered and it followed PRISMA guidelines

**Response:** Thanks for the comment
**Comment:** The inclusion criteria and exclusion criteria are very poorly written. Many sentences are incomplete and need major revision

**Response:** Thanks for the comment. The incomplete sentences have now been corrected and modified.

**Comment:** The authors can add a supplementary table regarding Newcastle-Ottawa Quality Assessment findings

Response: Thanks for the comment. Authors have now added a supplementary table
**Comment:** For the data synthesis, I would suggest the authors to recheck the last sentence which reads “to evaluate the effects of probable factors influencing study heterogeneity, the meta-regression test was performed.” I reckon there is some problem in the sentence

**Response:** Thanks for the comment: the sentence is rephrased. It now reads ‘’ Finally, meta-regression was performed to assessed the factors influencing the review’s heterogeneity’’.

Results:
**Comment:** The authors documented I-square value of 91% but did not describe the heterogeneity well. This high heterogeneity of the studies puts the findings of meta-regression into question. I would suggest the authors to describe the heterogeneity among the studies more elaborately. Secondly, I would suggest the authors to do a sub-group analysis with the studies that are more homogenous, and check whether it can replicate the overall result.

**Response:** First of all, the authors thank the reviewer for the recommendation. The presence of heterogeneity among studies was quantified by estimating variance using the I^2^ statistics. Thus, the pooled burden of UI was calculated using a weighted inverse variance random-effects model in order to adjust for this effect. Also, Sub-group analysis were performed with regards to sub-region which are Homogenous [ East africa vs.West Africa vs. South Africa], and setting [Hospital-based vs. Community-based studies]. The pooled estimate of UI in East Africa was 31% [95% CI: 19%-42%], West Africa was 16% [95% CI: 11%-21%], and South Africa 35% [95% CI: 15%-55%]. With regards to settings, the estimated burden of UI was 18% [95% CI: 13%-22%] for hospital based studies, and 23% [95% CI: 12%-33%] for community-based studies

**Comment:** The authors could describe the findings from associated factors more elaborately

**Response:** Thanks for the comment. The authors have now elaborated more on the associated factors for UI in SSA. The sentence reads ‘’ Systematic review of factors associated with UI amongst SSA women is shown Table 3. The significant independent factors were; parity(13,31,32), constipation(13,16,45), overweight/obese. (16,35,45), vaginal delivery(32,34,35) chronic cough (15,45), gestational age(13,35) and aging(15,32). For example, Berhe et al. found that multiparous women had approximately 6 times higher chances of developing UI compared to primigravida women in Ethiopia(13). A population-based study in rural Tanzania showed that women who had experienced multiple births had 2 times chance of reporting UI(31). Also, women with history of constipation had 2times chance of reporting UI compared to women without prior constipation in a Nigerian study(16). Similarly, an Ethiopian study reported history of constipation as an associated factor for UI (45). The results are presented in Table 3.
